# Complete genome sequence of *Bacillus licheniformis* KNU11, isolated from soil

**DOI:** 10.1128/mra.01011-24

**Published:** 2025-11-12

**Authors:** Minsoo Jeong, Min-Ji Kim, Jae-Ho Shin

**Affiliations:** 1 Department of Applied Biosciences, Kyungpook National University 34986, Daegu, Republic of Korea; 2 NGS Core Facility, Kyungpook National University 34986, Daegu, Republic of Korea; Rochester Institute of Technology, Rochester, New York, USA

**Keywords:** *Bacillus licheniformis*, complete genome, soil

## Abstract

The complete genome sequence of *Bacillus licheniformis* strain KNU11, a soil isolate that displays robust proteolytic enzyme production (e.g., proteases) and potential to degrade environmental pollutants. The 4,201,713 bp circular chromosome (46.0% G + C) encodes 4,154 proteins, providing insights into its metabolic versatility for industrial and agricultural applications.

## ANNOUNCEMENT

In this study, we report the complete genome sequence of *Bacillus licheniformis* strain KNU11, isolated from soil samples collected from farmland located in the greenhouse of Kyungpook National University, Daegu, Republic of Korea (GPS: 35.8944, 128.6124). This species is known for its diverse metabolic capabilities, including the production of proteolytic enzymes (e.g., proteases) (1) and the potential to degrade pollutants (2), and has been widely studied for its potential in various applications such as bioremediation (e.g., heavy metal removal) (3) and plant growth promotion (4). The strain was isolated through serial dilution (10-fold, 10^–1^ to 10^–5^) and plating on Luria-Bertani agar, followed by incubation at 30°C for 48 h under aerobic conditions (5). A single colony was selected and purified. This genome sequencing project was undertaken to explore the genetic potential of this strain for biotechnological applications.

Genomic DNA of *B. licheniformis* KNU11 was extracted using a phenol-chloroform extraction method based on the protocol described by Sambrook, Fritsch, and Maniatis in Molecular Cloning: A Laboratory Manual (Cold Spring Harbor Laboratory Press) (6). DNA quality and concentration were confirmed using the NanoDrop 2000 spectrophotometer (Thermo Fisher Scientific, Waltham, MA, USA) and Qubit 4 Fluorometer (Thermo Fisher Scientific). Sequencing was performed on the PacBio Sequel platform (Pacific Biosciences, Menlo Park, CA, USA), and the library was prepared using the SMRTbell Template Prep Kit (Pacific Biosciences) without DNA shearing. Size selection was performed using BluePippin (Sage Science, Beverly, MA, USA) to enrich for fragments larger than 10 kb. Sequencing employed P6-C4 chemistry, and the genome assembly was conducted using HGAP v4 (7), achieving a coverage depth of 714×. SMRT Link v7.0.0 (Pacific Biosciences) was used for read correction, trimming, and genome assembly, and contigs were bridged to form a single circular chromosome.

**Fig 1:**
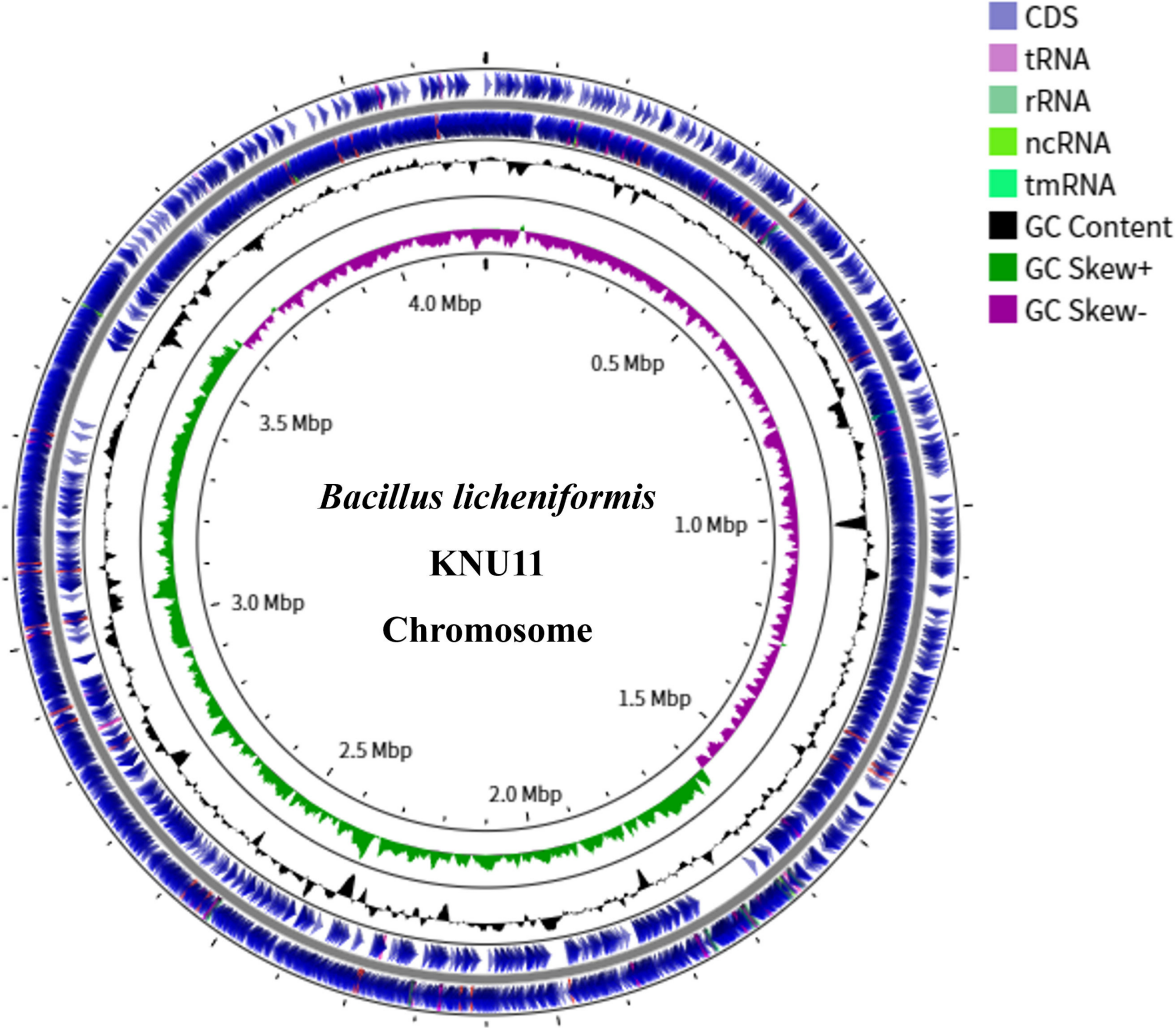
Circular genome map of *Bacillus licheniformis* KNU11. The genome map illustrates the complete circular chromosome of *B. licheniformis* KNU11. The inner rings display the distribution of coding sequences, rRNA, and tRNA genes, while the outer ring shows the scale in base pairs.

The complete genome of *B. licheniformis* KNU11 consists of a single circular chromosome of 4,201,713 base pairs with a G + C content of 46.0%. The assembly resulted in a fully closed circular genome, with an *N*_50_ of 4,201,713 bp. Genome annotation was performed using the NCBI Prokaryotic Genome Annotation Pipeline (8), which identified a total of 4,154 protein-coding genes, 81 tRNA genes, and 24 rRNA genes (Table 1). Additionally, 82 pseudogenes were annotated. Figure 1 shows a genome map of the circular chromosome of *B. licheniformis* KNU11, generated using CGView.

**TABLE 1:** Genetic feature of *B. licheniformis* KNU11

Feature	Value
Genome size (bp)	4,201,713
Number of contigs	1
G + C ratio (%)	46.0
Number of protein-coding genes	4,154
rRNA genes	24
tRNA genes	81
tmRNA genes	1
ncRNA genes	5
Pseudo genes	82

## Data Availability

The raw sequencing data for *Bacillus licheniformis* strain KNU11 have been deposited in the NCBI Sequence Read Archive (SRA) under the accession number SRR31210302, associated with BioProject PRJNA556772.
